# In vitro characterization of intrinsic properties and local synaptic inputs to pyramidal neurons in macaque primary motor cortex

**DOI:** 10.1111/ejn.14076

**Published:** 2018-08-04

**Authors:** Wei Xu, Stuart N. Baker

**Affiliations:** ^1^ Institute of Neuroscience Newcastle University Newcastle upon Tyne UK

**Keywords:** In vitro, motor cortex, primate

## Abstract

Primates (including humans) have a highly developed corticospinal tract, and specialized motor cortical areas which differ in key ways from rodents. Much work on motor cortex has therefore used macaque monkeys as a good animal model for human motor control. However, there is a paucity of data describing the fundamental functional architecture of primate primary motor cortex, which is best addressed with in vitro approaches. In this study we examined the cellular properties and the micro‐circuitry of the adult macaque primary motor cortex by carrying out in‐vitro intracellular recordings. We aimed to characterize the basic properties of the cortical circuitry by studying the intrinsic properties of its pyramidal neurons and their physiological interconnectivity. We studied the passive and active electrophysiological properties of pyramidal neurons in both superficial and deep cortical layers. Both superficial and deep pyramidal neurons exhibited bursting behaviour that could act as powerful excitation for downstream targets. Synaptic connections were lamina specific. Neurons in the deep layers had convergent excitatory inputs from all cortical layers whereas superficial neurons had only significant inputs from superficial layers. This sheds light on the functional architecture of the primate primary motor cortex and how its output is shaped. We also took the unique opportunity in our recording technique to characterize the relationship between intracellular and extracellular spike waveforms, with implications for cell‐type identification in studies in awake behaving monkey. Our results will aid the interpretation of primate studies into motor control involving extracellular spike recordings and electrical stimulation in primary motor cortex.

**Abbreviations**
ACSF: artificial cerebrospinal fluid
AHP: after hyperpolarization
ANOVA: analysis of variance
Em: membrane potential
EPSP: excitatory postysynaptic potential
ISI: interspike interval
i.v.: intravenous
M1: primary motor cortex
Ri: input resistance
*SEM*: standard error of mean

## 1 INTRODUCTION

The primary motor cortex (M1) in primates is the major effector of complex co‐ordinated voluntary movements such as precision grip and reaching (Baker, Pinches, & Lemon, 2003; Baker, Spinks, Jackson, & Lemon, 2001; Graziano, Taylor, & Moore, 2002). It receives numerous cortical and subcortical inputs (Holsapple, Preston, & Strick, 1991; Hoover & Strick, 1999; Huerta & Pons, 1990; Miyachi et al., 2006; Tokuno & Tanji, 1993), which provide information about higher motor commands, the current status of the motor system, sensory context and error signals. These inputs must be merged with pattern generators intrinsic to the cortex itself to produce useful patterns of motor output (Shenoy, Sahani, & Churchland, 2013). Processing within M1 necessarily depends upon both the intrinsic electrophysiological properties of its neurons and their interconnectivity. For example, the output of a corticospinal neuron depends both on the all‐or‐none inputs from presynaptic neurons, and how these are integrated within the dendritic trees.

Limited insights into intrinsic cell properties can be provided by surrogate measures taken from extracellular recordings. Action potentials recorded extracellularly are similar to a scaled version of the time derivative of the intracellular spike (Gold, Henze, Koch, & Buzsaki, 2006; Henze et al., 2000). In rodent cortex this allows putative separation of cortical interneurons from pyramidal neurons on the basis of spike width (Bartho et al., 2004), although in primate M1 such separation is impossible because fast‐conducting corticospinal cells have narrow spikes similar to those generated by interneurons (Vigneswaran, Kraskov, & Lemon, 2011). Information about the postspike after‐hyperpolarization can be yielded by statistical analysis of the interspike interval histogram distribution (Matthews, 1996). In monkey M1 and somatosensory cortex, this reveals that some cells exhibit a peak in their postspike trajectory, which biases firing to a preferred interspike interval (Wetmore & Baker, 2004; Witham & Baker, 2007). However, such methods are necessarily indirect, and many intrinsic properties such as membrane time constant, input resistance and the firing rate‐current relationship are unobtainable from extracellular records.

There are many available reports of intracellular recordings from rodent M1, both in vitro and in vivo—the latter have proliferated recently with the development of techniques for patch clamping in awake behaving rats (Lee, Manns, Sakmann, & Brecht, 2006). Fewer reports exist from species other than rodents, reflecting the highly challenging nature of such work. Studies in cat have examined pyramidal neuron intrinsic properties (e.g., Reyes & Fetz, 1993; Stafstrom, Schwindt, Flatman, & Crill, 1984). In monkey M1, spike triggered averaging of an intracellular recording from a simultaneous extracellular spike train has revealed synaptic inputs to pyramidal neurons (Matsumura, Chen, Sawaguchi, Kubota, & Fetz, 1996). However, the difficulty of locating recording sites within the complex, curved cortical surface precluded statements on the laminar identity of pre and post‐synaptic cells. The same group reported on the after‐hyperpolarization of monkey M1 neurons (Chen & Fetz, 2005), finding a subgroup of cells with peaked trajectories in agreement with the indirect extracellular measurements made by Wetmore and Baker (2004). The precarious nature of recordings, however, precluded full characterization of intrinsic properties.

A more detailed exploration of single cell parameters and local synaptic connectivity requires in vitro analysis. Because they have no pulsation movements, brain slice preparations are inherently more stable, permitting longer duration recordings of higher quality. It is also straightforward to identify the laminar location of electrodes. However, the high cost of animals means that this technique has not hitherto been applied to primate motor cortex. In this study, we present a characterization of intrinsic properties and local synaptic connectivity in M1 from macaque monkeys, the most commonly used primate species. We characterize the active and passive membrane properties of putative pyramidal neurons and find different patterns of input convergence between superficial and deep cells.

## 2 MATERIALS AND METHODS

All experimental procedures were carried out under the authority of personal and project licences issued by the UK Home Office, and were approved by the Animal Welfare and Ethical Review Board of Newcastle University. Treatment of animals complied with European Directive 2010/63.

### 2.1 Monkey anaesthesia

Terminal experiments were performed on 12 female and six male rhesus macaques (*M. mulatta*) ranging from 4 to 9 years old, obtained from the Health Protection Agency, UK and MRC Centre for Macaques, UK. All animals were either at the end of unrelated studies in vivo, or were about to be culled as part of the management of the breeding colony from which they came. Two monkeys had previously been used for long‐term in vivo experiments, involving single unit recordings from other brain areas in the awake state. Monkeys were sedated with intramuscular ketamine (10 mg/kg) before anaesthesia induction with either i.v. propofol or inhaled sevoflurane. They were then intubated and ventilated with 2.5%–3.5% sevoflurane or desflurane in 100% oxygen. Single i.v. doses of buprenorphine (20 μg/kg) and meloxicam (0.3 mg/kg) were given prior to head fixation and an i.v. infusion of methylprednisolone (5.4 mg/kg/hr) was established to minimize cerebral oedema during surgery. Monitoring included pulse oximetry, heart rate, noninvasive blood pressure, core and peripheral temperature, and end‐tidal carbon dioxide concentration. The animal was kept warm with a thermostatically controlled heating blanket, as well as a separate system which surrounded the body in warm air. Maintenance i.v. fluids were given throughout the procedure (Hartmann's solution, 10 ml/kg/hr). After M1 tissue removal the monkeys were transcardially perfused with ice cold sucrose Ringer and allowed to exsanguinate via an incision in the right ventricle. During the perfusion neuronal tissue was removed from the brainstem, spinal cord, and the cerebellum for unrelated in vitro experiments.

### 2.2 Slice preparation

Bilateral craniotomies were made to expose the area of the central sulcus and precentral gyrus and the cortical surface was doused in ice‐cold sucrose Ringer solution (constituents in mM: 252 Sucrose, 3 KCl, 1.25 NaH_2_PO_4_, 1 MgSO_4_, 1.2 CaCl_2_, 10 Glucose, 24 NaHCO_3_) before a block of tissue was excised using a scalpel blade, and lifted into ice‐cold sucrose Ringer using a spatula. The medial border of the block of tissues was the sagittal fissure. The lateral border was ~2 cm lateral to the sagittal fissure. The anterior border was ~1 cm anterior to the central sulcus. The posterior border was just posterior to the central sulcus to include a small part of the primary somatosensory cortex for the purpose of orienting the slice. All recordings were done from the precentral gyrus. Parasagital 450‐μm‐thick slices were rapidly prepared on a VF‐300 vibratome (Precisionary Instruments Inc, Greenville, North Carolina, USA) in ice‐cold sucrose Ringer. Each block of tissue produced around 20 slices. Slices were then transferred into an interface chamber containing artificial cerebral spinal fluid (ACSF—same constituents as sucrose Ringer apart from sucrose being replaced by 126 mM NaCl) and held at room temperature for at least 1 hr before recording. All solutions were constantly bubbled with a 95% O_2_ and 5% CO_2_ gas mixture. Slices were recorded in an interface recording chamber (model BSC‐ZT, Harvard Apparatus, Cambridge, UK) whilst superperfused with ACSF. Humidified gas of 95% O_2_ and 5% CO_2_ flowed over the slice surface. The chamber was thermostatically held at 32–33°C. After M1 tissue was removed, the animal was perfused transcardially with sucrose Ringer and samples of brainstem and spinal cord removed for other, unrelated in vitro studies.

### 2.3 Recording

Electrodes were pulled from borosilicate glass capillaries on a model P‐1000 Flaming/Brown micropipette puller (Sutter Instruments, Novato, California, USA). Electrodes were filled with 2 M potassium acetate and 2% Biocytin and their impedance values ranged from 100 to 150 MΩ. Where possible, after recordings were complete cells were filled with Biocytin using repetitive hyperpolarizing current pulses (alternating 0.5 s long positive and negative square wave current injections at 0.2 nA for at least 20 min). Slices were then placed in 4% paraformaldehyde; labelled cells were subsequently stained using a standard Vectastain ABC kit (Vector Laboratories, Peterborough, UK).

Two pipette electrodes were used simultaneously to increase yield. Pipettes were located by approximately aligning them with contacts 3–6 or 12 of the 16 contact silicon probe (see below). This produced penetrations 200–500 μm or 1,100 μm from the pial surface, which corresponds approximately to cortical laminae II/III and V respectively (Lacroix et al., 2004; Matelli, Luppino, & Rizzolatti, 1985; Shepherd, 1998). Each electrode was mounted on a piezoelectric motor (NanoPZ Ultra‐High resolution actuator, Newport). Voltage recordings and current injections was carried out using a BA‐03X bridge amplifier (NPI Electronics, Tamm, Germany) with ×10 gain and low‐pass filter set to 10 kHz. Parasitic capacitance transients were compensated for and the bridge was checked and balanced regularly. Custom‐made software (Collins & Baker, 2014) controlled the piezoelectric motors directly and monitored electrode voltage readings and injected current through the electrodes via the digital and analogue input–output functions of USB National Instruments data‐acquisition device (USB‐6356 X‐series, National Instruments, Austin, Texas, USA). The software advanced the electrodes automatically and simultaneously to find the surface of the slice, then moved both electrodes together automatically in 2 μm steps until it detected a voltage drop of more than 30 mV. Movement on both pipettes was then stopped to allow the user to ascertain whether a neuron had been impaled. Data were digitized using a Micro1401 interface at 25 kHz and recorded using Spike2 software (Cambridge Electronic Design, Cambridge, UK).

Recordings were either targeted at cortical layers II/III or layer V based on the results of previous histological studies (Lacroix et al., 2004; Shepherd, 1998).

### 2.4 Measurement of intrinsic properties

The electrode's bridge balance was continuously monitored and adjusted to compensate for the electrode resistance. Each cell's input resistance (*R*_i_) was calculated from averaged traces of small voltage deflections (<−10 mV from resting membrane potential, *E*_m_) to small square hyperpolarizing currents (1 s in duration). Membrane time constants (τ) were calculated from these responses by generating semi‐logarithmic plots of the initial part of the voltage deflection, and fitting these with linear regressions.

The threshold voltage for spontaneous action potentials was defined as the membrane potential at which its derivative first exceeded 50V/s (Kole & Stuart, 2008). The absolute ratio of the maximum derivative divided by the minimum derivative was used as a measure of the asymmetry of the spike waveform (McCormick, Connors, Lighthall, & Prince, 1985). Spike half widths were measured at the halfway voltage value between threshold voltage and peak spike voltage. The time of the spike after hyperpolarization (AHP) was defined as the time to reach the most hyperpolarized voltage value after the spike peak; the AHP depth was defined as the difference between threshold voltage and this voltage minimum.

Action potentials were discriminated offline using the Spike 2 software. In order to analyse bursting behaviour, an evoked burst was defined as three or more consecutive spikes at the start of an evoked spike train with interspike intervals all below half of the mean interspike interval in the train. The duration of the burst, *T*, was then defined as the sum of all intervals in the burst. The number of spikes in the burst, *n*, is defined as the number of spikes that make up all the burst interspike intervals minus one. The Poisson surprise index, *S*, as developed by Legendy and Salcman (1985) was used to quantify the degree of bursting for spike trains that contained a burst. This is essentially a measure of the unlikelihood of having *n* or more number of spikes in an interval of duration *T* if the expected spike rate is *r*:

(1) S=−logP

where

(2) $$P=1-e^{-rT}\sum_{i\;=\;0}^{n-1}{\left(rT\right)}^{i}/i!$$

### 2.5 Extracellular stimulation

A silicon probe with 16 parallel shanks 100 μm apart, and one contact at the tip of each shank (A1x16, NeuroNexus, Ann Arbor, Michigan, USA) was used to deliver extracellular stimuli. The row of shanks was placed perpendicular to the slice cortical surface with the first contact resting on the surface; this arrangement spanned 1.5 mm of cortical depth. The lateral distance between the probe and the intracellular recording pipette was ~0.5 mm. The probe was connected to a custom circuit which used relays to switch one probe contact at a time to an isolated stimulator (model 2100, A‐M systems Inc, Sequim, Washington, USA). Biphasic stimuli (0.1 ms per phase; 20–100 μA) were delivered with an interstimulus interval of 100 ms. Contacts were stimulated in pseudo‐random order, with a 10 ms delay between relay activation and delivery of the stimulus to ensure that the relay contacts had stabilized. Typically 100–200 stimuli were given to each contact, although sometimes smaller stimulus numbers were available for analysis if a cell was lost prematurely.

Stimulus responses were analysed differently depending on whether spikes were evoked. In cells which did not spike in response to the probe stimuli, each stimulus contact was first tested to see if it evoked a significant depolarization (i.e., an EPSP). The time of the peak in averaged voltage waveform was noted for each contact and the voltage at that time for each individual trial against the voltage value near the end of that trial (at 70 ms post‐stimulus, when any EPSP would have decayed back to resting potential) was compared using a paired *t*‐test. Because using the time of the maximum averaged voltage necessarily introduced a bias for higher than average voltage values for each trial, we used a significance level of 1% (instead of the commonly used 5%). The amplitude of the EPSP was calculated as the difference between the peak voltage and the voltage near the end of the trial (70 ms post stimulus).

In cells which did show spikes following the probe stimuli, peri‐stimulus time histograms were compiled, and the average number of spikes evoked per stimulus in the first 10 ms was measured; this window was chosen to minimize the inclusion of spontaneous spikes.

All measures are quoted in the Results section as mean ± *SEM* unless otherwise stated.

## 3 RESULTS

**Figure 1:**
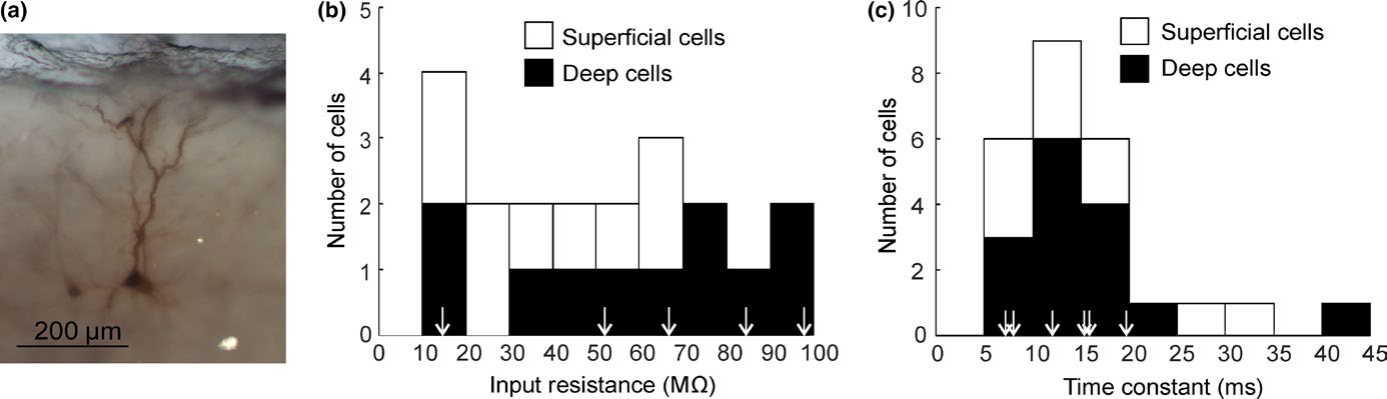
Passive neuronal properties. (a) Labelled pyramidal neuron. (b) Histogram of the distribution of membrane input resistance. (c) Histogram of the distribution of membrane time constants. In (b) and (c), the values measured from neurons successfully filled and subsequently identified as pyramidal neurons are indicated by arrows [Colour figure can be viewed at http://www.wileyonlinelibrary.com/]

Intracellular recordings were made from a total of 189 neurons from the precentral gyrus, of which 90 were useable (giving an average of five neurons per animal). The total number of cells penetrated per animal varied from 0 to 26. Of the available cells, 64 were characterized as deep, that is, within layer V, and 26 as superficial (layer II/III) based on depth measurements from the cortical surface. Not all cells were held for sufficient time to allow all tests to be completed; numbers contributing to each measurement are given individually below. Nine cells were injected with biocytin and then successfully stained subsequently; this revealed all nine neurons to be pyramidal neurons (four deep, five superficial; see example in Figure 1a).

### 3.1 Passive membrane properties

A short train of injury discharges was invariably seen after cell impalement by the electrode. This initial discharge tended to settle down to a slower rate or cease entirely. Only cells that settled to a stable membrane potential (*E*_m_) below −50 mV were used for characterization. Mean resting *E*_m_ was −60.2 ± 2.2 mV (*n* = 21). Input resistance was measured in 18 cells (nine superficial and nine deep) and membrane time constant in 25 cells (10 superficial and 15 deep) and found to be, respectively, 45.3 ± 6 MΩ and 15 ± 1.6 ms. The distribution of these parameters are plotted in Figure 1b,c; they did not differ significantly between deep and superficial neurons. These values are in broad agreement to those in nonprimate pyramidal neurons measured using similar sharp micropipette electrodes (Connors, Gutnick, & Prince, 1982; McCormick et al., 1985; Nowak, Azouz, Sanchez‐Vives, Gray, & McCormick, 2003) and of similar ranges to primate layer V pyramidal neurons investigated using whole‐cell recordings (Chang & Luebke, 2007; Luebke & Chang, 2007).

### 3.2 Action potential waveform

**Figure 2:**
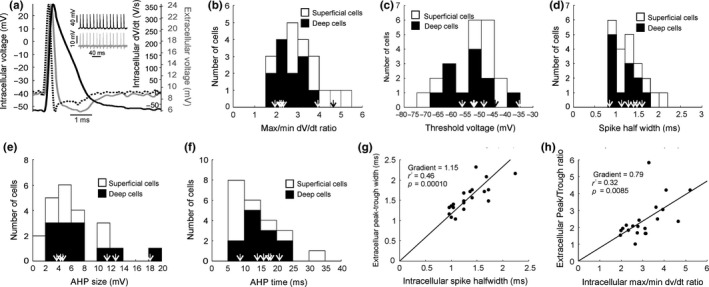
Action potential properties. (a) Averaged waveform of an action potential (black), its first derivative (dashed) and the simultaneously recorded extracellular waveform (grey). Example raw data on a longer timescale is shown in the inset. Histograms showing the distribution of max/min dv/dt ratios (b), threshold voltages (c), spike half widths (d), AHP sizes (e) and AHP latencies (f). In all cases, values measured from cells subsequently filled and successfully visualized as pyramidal neurons are indicated by arrows. The number of cells per interval is indicated by the highest point of the bin; each bin is then split into two colours to indicate how many cells in that bin comes from superficial and how many from deep layers. (g) Relationship between intracellular spike half width and extracellular spike peak‐to‐trough duration. (h) Relationship between intracellular max/min d*v*/d*t* ratio and extracellular peak/trough ratio. In (g, h), scatter plots are overlain with a linear regression line forced through the origin

For each cell the averaged action potential waveform was compiled from at least ten spontaneous action potentials triggered from threshold (see example in Figure 2a, black trace). The waveform was then numerically differentiated (red line, Figure 2a). From these two traces, various parameters were measured and shown in Figure 2b–f.

The average absolute ratio of the maximum to minimum derivative was 3.0 ± 0.2; spike threshold was −52.6 ± 2 mV; spike half width was 1.27 ± 0.07 ms; AHP time was 13.4 ± 1.4 ms; and AHP depth was 6.4 ± 0.9 mV (*n* = 22 for all). Distribution histograms for these measures are shown in Figure 2b–f. The derivative ratio was similar to values reported in guinea pigs for bursting pyramidal neurons, but larger than for interneurons (McCormick et al., 1985). The distribution of other measures was compatible with prior studies on pyramidal neurons. In addition, for all measures the cells which had been labelled and visually identified definitively as pyramidal neurons had values comparable to the population as a whole. It is therefore likely that the neurons reported in this paper are overwhelmingly pyramidal neurons; this is compatible with a pyramidal neurons forming ~70% of the neocortical neuronal population and the probable recording bias towards the larger pyramidal neurons when using sharp penetrating micropipette electrodes (Shepherd, 1998). Some of the histograms appeared to have a bimodal distribution (e.g., Figure 2c, deep cells in Figure 2d). We explored whether these peaks might reflect bursting versus regular spiking cells, but found no consistent differences in burst index between members of the subpeaks for both Figure 2c (superficial and deep cells, *p* = 0.32, unpaired *t*‐test between two groups for burst index measured at 0.4 nA current injection) and Figure 2d (deep cells only, *p* = 0.87, unpaired *t*‐test between two groups for burst index measured at 0.4 nA current injection).

Our automated recording set up used two nearby micropipette electrodes which were advanced simultaneously to increase the recording yield. An unintended benefit of this arrangement was that, on 20 occasions, we recorded an extracellular spike waveform on one electrode after penetrating the cell with the other (Figure 2a, blue trace). The magnitude of the extracellular action potentials ranged from 1.1 to 17.2 mV depending on how close together the two electrodes were. These electrodes had very similar impedances and identical signal filtering, providing an important opportunity to examine what the extracellular spike waveform can tell us about the intracellular action potential. In agreement with previous results (Gold et al., 2006; Henze et al., 2000) the extracellular waveform broadly resembled the shape of the first derivative of the intracellular waveform, although the peak‐to‐trough width of the first derivative was on average significantly smaller (1.12 ± 0.09 ms vs. 1.57 ± 0.08 ms, *p* = 0.00063 paired *t*‐test). Moreover the intracellular spike half‐width was significantly correlated with the extracellular peak‐to‐trough duration (Figure 2g, *r* ^2^ = 0.46, regression constrained to pass through the origin, *p* = 0.00010).

The ratios of the maximum to minimum of the derivative of the intracellular waveform were also plotted against the extracellular waveforms’ peak/trough ratios (Figure 2h). Although there was a significant positive correlation between these measures, there was considerable variability around the regression line (*r* ^2^ = 0.32; *p* = 0.0085).

One possible explanation for the low correlation coefficients found in both of these analyses is that the extracellular action potentials varied considerably in amplitude, and hence in signal to noise ratio. To check for this, we divided the dataset into two groups, depending on whether the extracellular action potential was above or below the mean amplitude. Correlations as shown in Figure 2g,h were then recomputed. In both cases the correlation was similar for smaller spikes and larger spikes (intracellular spike half‐width vs. extracellular peak‐to‐trough duration: *r* ^2^ = 0.60 and 0.68; intracellular derivative maximum to minimum ratio vs. extracellular peak to trough ratio: *r* ^2^ = 0.40 and 0.46). We therefore conclude that different signal to noise ratios of the extracellular action potential cannot explain the low correlations. Variability may be introduced by different filtering properties of the extracellular matrix depending on the (here uncontrolled) distance between the extracellular electrode and the neuron. The uncontrolled distance to the recorded cell simulates the scenario during extracellular in vivo recording. Given these correlation coefficients, the extracellular action potential parameters cannot be used unequivocally to derive intracellular waveforms, and therefore should not be used on their own to distinguish between cells of different types in primate M1 (Kaufman et al., 2010; Merchant, Naselaris, & Georgopoulos, 2008; Mitchell, Sundberg, & Reynolds, 2007; Vigneswaran et al., 2011).

### 3.3 Frequency responses to injected square current pulses

**Figure 3:**
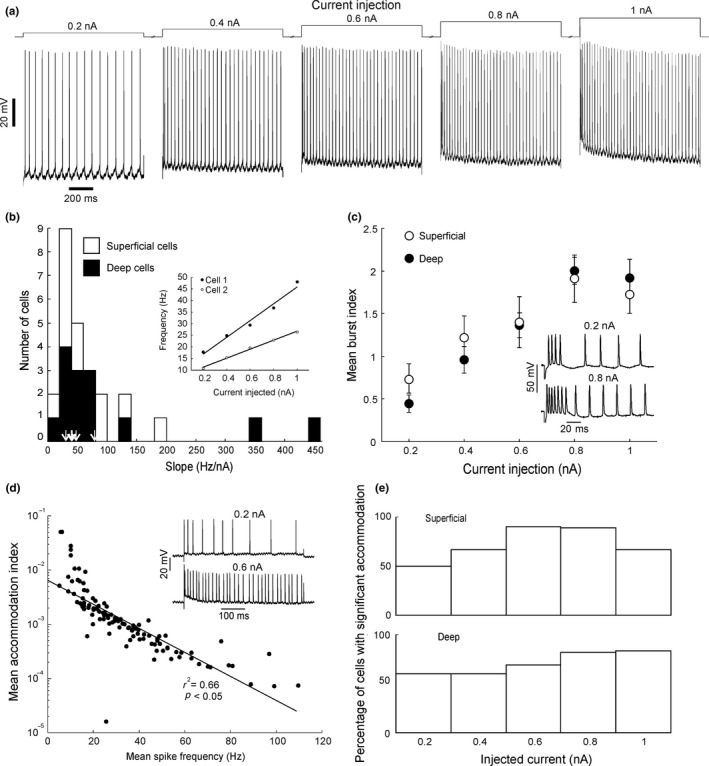
Spiking responses to current injection. (a) Example spike trains evoked by different current injections. (b) Histogram of distribution of gradients of linear regressions for frequency versus current relationships for all cells tested. Values of labelled pyramidal neurons (5 cells) are indicated by arrows. Inset shows two examples of relationship between current injected and mean (±*SE*) frequency of evoked spike trains. Straight lines represent linear regressions. (c) Mean and *SE* of burst indices (see Section 2) for all cells plotted against current injection. Inset shows example of bursting behaviour of a neuron to two different levels of current injections. (d) Mean accommodation index for each cell plotted against the mean frequency of the evoked spike train on a semi‐logarithmic scale. Inset shows example raw data for two levels of current injection. (e) Bar chart of percentage of superficial and deep cells with significant accommodation

**Figure 4:**
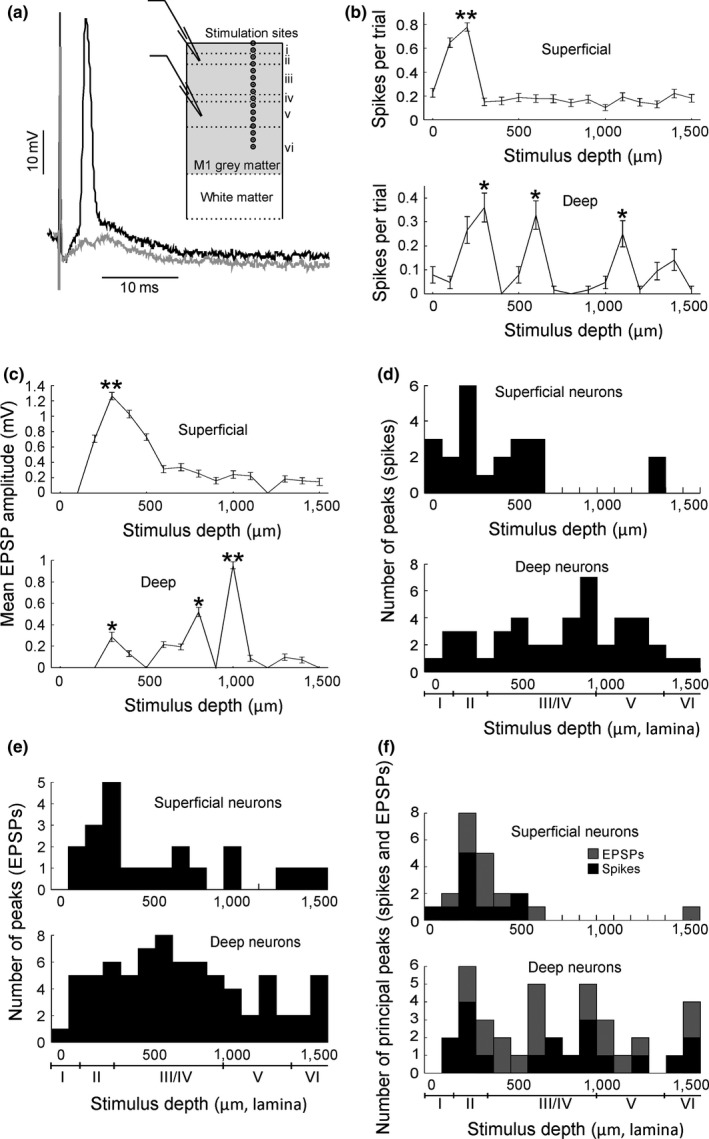
Responses to extracellular stimulation. (a) Two sample traces of a spike (black) and an EPSP without spike (grey) elicited by extracellular stimulation. Inset shows scheme of recording setup. (b) Sample responses from two spiking neurons to electrical stimulation of different cortical depths. (c) Sample responses from 2 nonspiking neurons to electrical stimulation of different cortical depths. (d) Histograms of significant spiking response peaks for superficial and deep spiking cells in response to stimulation of different cortical depths. (e) Histograms of pooled significant EPSP response peaks for superficial and deep nonspiking cells. (f) Histogram of pooled principal peaks for superficial and deep cells for both spiking and nonspiking cells. Equivalent lamina depths underneath histograms are taken from Shepherd (1998). (b) Top panel: *p* = 5.65e‐24; bottom panel: *p* = 1.22e‐80; One‐way ANOVA test. (c) Top panel: *p* = 6.57e‐85; bottom panel; *p* = 1.14e‐221; One‐way ANOVA test. *Stimulus contacts that are significantly higher both neighbouring contacts. **Principal peak ‐ stimulus contacts that are significantly higher than all other contacts

Positive square current pulses (1 s duration; amplitude 0.2 to 1 nA) were injected to assess spiking responses. In all healthy neurons tested such currents evoked trains of spikes (see example in Figure 3a). The average frequency of the spike train tended to increase in a linear fashion with increasing current amplitude (linear regression *r* ^2^ = 0.91 ± 0.26; two example cells shown in Figure 4b inset). The mean slope of the frequency‐current relationship was 83.7 ± 20.2 Hz/nA (*n* = 26; distribution histogram presented in Figure 3b). These slopes are in the same range as those measured in regular spiking pyramidal neurons from cat visual cortex (Nowak et al., 2003).

Pyramidal neurons in rats, guinea pigs and cats have been separated into “regular spiking” and “bursting” cells (Agmon & Connors, 1989; Chagnac‐Amitai, Luhmann, & Prince, 1990; McCormick et al., 1985; Nowak et al., 2003; White, Amitai, & Gutnick, 1994). Some of the macaque M1 neurons also showed a tendency to fire a high‐frequency burst of spikes at the beginning of the evoked spike train (example in Figure 3c inset). It was sometimes difficult to tell by eye whether there was an initial burst or whether the initially relatively short interspike intervals were part of an accommodating spike train. Therefore an objective measure of the presence or absence of a burst and the degree of bursting was used. A burst was defined as three or more consecutive spikes at the start of the spike train with their interspike intervals smaller than half the mean interspike interval in the train. Based on this criterion 22/27 cells showed an initial burst to at least one level of current injection. Bursting cells were seen in both deep (15 cells) and superficial (12 cells) layers. The degree of bursting for a given current amplitude was quantified using the Poisson surprise index (see Section 2; Legendy & Salcman, 1985). This measure takes into account both the duration and frequency of the burst. It tended to increase with increasing current injection up to 0.8 nA, after which it fell because the mean spike frequency of the train became comparatively too high; pooled data across all 27 cells are plotted in Figure 3c.

Similar to nonprimate pyramidal neurons, some evoked spike trains in macaque M1 neurons tended to slow down their discharge rate during the injected current pulse (e.g., in Figure 3d inset). Spike rate accommodation was quantified for each individual spike train by calculating the mean consecutive differences of interspike intervals, termed the “accommodation index.” This measure is relatively insensitive to the initial evoked bursting. A positive accommodation index indicates a trend for increasing ISIs during the current pulse. The accommodation index tended to decrease with increasing current injection (Figure 3d). The mean accommodation index for each current intensity across all cells are pooled and plotted against average evoked spike train frequency in Figure 3e. The semi‐log plot suggests a roughly exponential relationship between spike frequency and accommodation with a decay constant of 18.2 Hz (5% confidence limits of 15.9–21.2 Hz; the value of 18.2 does not sit centrally between the confidence intervals because it is derived from the confidence interval of the straight line fit of the semilog plot). In order to test the significance of accommodation the interspike intervals for each evoked spike train were randomly shuffled 1,000 times and the accommodation index for each shuffled train was ranked. The 950th highest accommodation index was taken to be the 5% significance threshold. The percentage of significant accommodating evoked spike trains is plotted in Figure 3e versus injected current, for superficial and deep cells. There was no significant difference between superficial and deep cell values across the range of injected currents (*p* = 0.61 paired *t*‐test).

### 3.4 Extracellular stimuli

In order to investigate the pattern of interlaminar connectivity, intracortical microstimuli were delivered through a 16‐shank silicon probe (shanks 100 μm apart), which was positioned on the brain slice so that the line of contacts was perpendicular to the cortical surface. In all cases, responses to stimuli were overall excitatory, that is, there was a net membrane depolarization. It is likely that inhibition was superimposed on excitation in some cases, potentially reducing the amplitude and duration of the depolarization, although we did not examine this further. Excitatory responses consisted of either an EPSP or an EPSP with one or more action potentials superimposed (see examples in Figure 4a). The presence of action potentials made quantification of EPSP amplitude unreliable; we accordingly took two different approaches to evaluating response size. Where no spikes were generated, we measured EPSP amplitude; if some stimuli‐elicited spikes, we instead measured the average number of evoked spikes per stimulus (see Section 2).

Examples of how response magnitude varied with the depth of the stimulus are shown for two superficial and two deep neurons in Figure 4b,c. Cells in Figure 4b fired spikes, those in Figure 4c did not, allowing us to illustrate the two approaches to response quantification. In some cases, all stimulus locations evoked responses; however there was usually a location that evoked a significantly larger response than the others. A one‐way ANOVA test and post hoc pair‐wise comparisons were performed for all possible pairs of stimulus locations for each cell. Locations which had responses significantly larger than the sites located either side of it were denoted as response peaks (marked by * in Figure 4b,c). Where a response peak was significantly larger than responses from all other stimulus locations (determined by individually comparing that response with the responses from the other 15 sites) then that peak was denoted as the “principal peak” (denoted by ** in Figure 4b,c).

The remainder of Figure 4 presents analysis of responses across the population of recorded neurons. Figure 4d shows counts of the number of significant responses, for instances where spikes were elicited; superficial and deep neurons are plotted separately. Figure 4e presents the equivalent histograms for cells which did not fire spikes in response to stimuli. Figure 4f shows the locations of just the principal peaks. These plots show that superficial cells tend to receive input from superficial stimulus sites whereas deep neurons receive input over a wider range of cortical depths.

The mean extracellular stimulus intensity was 43.1 ± 2.2 μA, ranging from 20 to 100 μA—same order of magnitude as those used in in vivo intracortical microstimulation (Baker, Olivier, & Lemon, 1998; Kraskov, Prabhu, Quallo, Lemon, & Brochier, 2011). For each cell the stimulus intensity was increased until at least one contact evoked either an EPSP or a spike consistently over a number of trials. For each cell the largest EPSPs had magnitudes of 2.84 ± 1.21 and 2.45 ± 0.38 mV, evoked in superficial and deep nonspiking cells respectively. The largest response evoked in superficial and deep spiking cells was, respectively, 0.30 ± 0.05 spikes/trial and 0.48 ± 0.07 spikes/trial.

## 4 DISCUSSION

The purpose of the study was to quantify intrinsic electrophysiological properties and functional connectivity within the adult macaque primary motor cortex.

### 4.1 Comparisons with nonprimates

Broadly speaking the passive properties of macaque pyramidal neurons were similar to those in nonprimates. However whereas rodent bursting pyramidal neurons tend to be located in layer 5 (Connors & Gutnick, 1990; Connors et al., 1982; Franceschetti et al., 1995; McCormick et al., 1985; Williams & Stuart, 1999), in the macaque M1 bursting neurons were frequently seen both in the superficial and deep layers. Burst firing has been variously attributed to voltage sensitive calcium conductance (McCormick et al., 1985; Williams & Stuart, 1999) and persistent sodium conductance (Franceschetti et al., 1995; Mantegazza, Franceschetti, & Avanzini, 1998) and has been postulated to enhance the strength of synaptic transmission and facilitate synchronization of large assemblies of neurons (Williams & Stuart, 1999). The presence of these neurons in the superficial layers of the primate M1, coupled with the largely unidirectional excitation from superficial to deep layers suggests the presence of a powerful system of top‐down excitation within the layers of primate M1.

### 4.2 Interlaminar connectivity

Models of cortical circuitry have all agreed on the columnar organization of the cortex but differ slightly on the functional interconnectivity between different laminae. Anatomical studies have revealed reciprocal projections between pyramidal neurons in layers II/III and layer V (Gilbert & Wiesel, 1979; Lund, Henry, MacQueen, & Harvey, 1979; Martin & Whitteridge, 1984) and functional models of cortical circuitry have taken this into account (Douglas & Martin, 2004, 2007). However these models of recurrent excitation between deep and superficial layers (derived mostly from the visual cortex) seem to be at odds with the functional circuitry of macaque M1 revealed in the present study and also in mouse M1 (Weiler, Wood, Yu, Solla, & Shepherd, 2008). Our results do not necessarily contradict anatomical findings, as some weak responses in both superficial and deep neurons could be evoked from stimuli in all layers. However, inputs to putative layer II/III neurons were strong only from the same layers, whereas by contrast strong inputs to layer V cells came from a wide range of depths.

It is known that there are more direct excitatory synapses from layers II/III to layer V than in the opposite direction (Binzegger, Douglas, & Martin, 2004), and our findings are thus in agreement with known anatomy. A further reason for the relatively weak deep‐to‐superficial excitation could be due to the thin layer IV in the primary motor cortex. The stimuli in our study may have activated not just local intracortical fibres, but also those from distance sources such as the thalamus. Thalamocortical axons terminate mainly in layer IV, which then projects to layers II/III, potentially providing an indirect deep‐to‐superficial excitatory pathway. Although layer IV in the primary motor cortex has been shown to receive thalamocortical projections (Barbas & Garcia‐Cabezas, 2015), this layer is scant compared to sensory cortices, which would reduce the opportunity for indirect deep‐to‐superficial excitation.

A general principal of cortical organization is that cortico‐cortico connections arise from layer II/III, whereas output to subcortical regions (including to the spinal cord, via the corticospinal tract) comes from layer V. Since the major function of M1 is to produce output to subcortical structures to generate movement, it might be expected that there would be a high convergence of excitatory inputs onto layer V neurons from all layers. It is also perhaps unsurprising that the strongest excitation to layer II/III neurons come from the same layers: previous work in visual cortex suggests that 70% of excitatory input to layer II/III pyramidal neuron comes from other pyramidal neurons within the same layer (Douglas & Martin, 2007). What is worthy of note is that, contrary to quantitative cortical models based on the visual cortex (Binzegger et al., 2004), there does not seem to exist strong excitatory feedback from layer V back to layers II/III. Interconnections between superficial and deep layers are thus highly asymmetric with superficial‐to‐deep excitation being much greater than deep‐to‐superficial. This is in agreement with the top‐down interlaminar excitation for mouse M1 (Weiler et al., 2008) but at odds with the reciprocal excitation between superficial and deep layer pyramidal neurons of the canonical cortical circuitry proposed for the cat primary visual cortex (Douglas & Martin, 1991).

The extracellular stimuli which we used were of low intensity (20–100 μA). Based on the results of Stoney, Thompson, and Asanuma (1968), we estimate current spread to be between 120 and 280 μm; we can therefore have confidence that effects were mediated by activation of elements close to the stimulating electrode. It is likely that some of the synaptic potentials originated from a monosynaptic pathway, involving direct activation of fibres or cells near the stimulating electrode which then projected to the recorded neuron. More complex oligosynaptic pathways are also likely to have contributed, for example involving indirect (transsynaptic) activation of cells in the same layer as the stimulating electrode, which then projected monosynaptically to the recorded neuron. We did not attempt to distinguish these many possibilities. Firstly, EPSP onset latency was sometimes occluded by the stimulus artefact sufficiently to make determination of the earliest component unreliable. Second, the slices were maintained at 33–34°C; this is lower than physiological temperatures, which would increase synaptic and axonal conduction delays beyond typical in vivo values in ways that are hard to estimate, blurring the distinction between expected mono‐ and di‐synaptic latencies. Instead, we accept that most responses were likely to be comprised of overlapping potentials mediated by multiple pathways. Nevertheless, the presence or lack of responses is still good evidence for the presence or absence of connections between neural elements close to the stimulating electrode and the recorded cell, which was the main focus of our study.

### 4.3 Implications for in‐vivo M1 recordings and stimulation

Intracortical microstimulation studies have hitherto focused on how effects change along the planar dimension on the cortical surface rather with depth (Boudrias, McPherson, Frost, & Cheney, 2010; Fitzsimmons, Drake, Hanson, Lebedev, & Nicolelis, 2007; Gioanni & Lamarche, 1985; Neafsey et al., 1986). Given the changes in interlaminar interconnectivity which we observed, it is reasonable to predict that different effects may be produced from focal stimulation of different cortical layers. Our recordings were made at only single points, relatively close to the stimulation array. It is known that M1 cells can be activated transynaptically by stimuli delivered several millimetres away (Baker et al., 1998; Hao, Riehle, & Brochier, 2016). This is the case for identified pyramidal tract cells, which are located in layer V, when stimuli are also given to layer V (Baker et al., 1998). We do not know how the spatial spread of excitation differs for the superficial‐to‐deep, and superficial‐to‐superficial connections which we have observed.

Extracellular recording in awake animals has provided much information on the functional role of M1 in motor control, but one problem with this method is the limited information which it can provide on neuron type. Whereas projection cells such as pyramidal tract neurons can be straightforwardly identified by antidromic activation (Baker et al., 1999), cells which cannot be so activated are typically grouped together as “unidentified cells” (Kozelj & Baker, 2014). Unlike in rodents, in monkey M1 spike width cannot reliably distinguish interneurons from pyramidal cells (Bartho et al., 2004; Vigneswaran et al., 2011). Our results indicate that although there is a correlation between parameters measured from extracellular and intracellular spikes of pyramidal cells, it explains less than 50% of the variance (Figure 2g,h). This makes it unlikely that separation of different cortical cell types would be possible on the basis of spike shape alone. It remains possible that recordings will be separable using more complex combinations of multiple measurements taken from the extracellular discharge, an approach which we have recently used successfully in the primate reticular formation (Zaaimi, Soteropoulos, Fisher, Riddle, & Baker, 2018).

## CONFLICT OF INTEREST

The authors declare no conflicts of interest.

## DATA ACCESSIBILITY

The supporting data for the article can be accessed upon request.

## AUTHOR CONTRIBUTIONS

W.X. carried out experiments and designed and carried out analysis. S.N.B. designed the experiments; designed and manufactured automated recording system, and designed the analysis methods.
