# Supplementary material for: In vitro characterization of intrinsic properties and local synaptic inputs to pyramidal neurons in macaque primary motor cortex
Source: Eur J Neurosci. 2018 Aug 4;48(4):2071–83. doi: 10.1111/ejn.14076 (PMC6175011; doi:10.1111/ejn.14076)
Supplement: Supplementary file 1 [file EJN-48-2071-s001.docx]

In Vitro Characterisation of Intrinsic Properties and Local Synaptic Inputs to Pyramidal Neurons in Macaque Primary Motor Cortex

*Xu, Wei;* Baker, Stuart

_____________________________________________________________________________________

| Review timeline: | Submission date: 12.04.18  Editorial Decision: Minor Revision  Revision Received:20.06.18  Editorial Decision: Minor Revision  Revision Received: 29.06.18  Accepted: 04.07.18 |  |
| --- | --- | --- |
|  |  |  |

Editor: John Foxe

Reviewer 1: Atsushi Nambu

Reviewer 2: Thomas Brochier

_____________________________________________________________________________________

1st Editorial Decision	16.05.2018

__________________________________________________________________________________ In Vitro Characterisation of Intrinsic Properties and Local Synaptic Inputs to Pyramidal Neurons in Macaque Primary Motor Cortex

Dear Dr. Xu,

Your manuscript has been reviewed by two external reviewers as well as by the Editorial Team.

As you can see, both reviewers consider this to be an important study in which you have generated a unique data-set that will be of great value to others in the field.  Both, however, raise a few points that need to be addressed in a revised version of the manuscript.  Reviewer 1 suggests including a diagram of the experimental setup and suggests clarification/expansion of the text in a number of places.  Reviewer 2 is primarily concerned about over-interpretation of the data suggesting that you tone down some of the conclusions.   Please carefully address each of the points that they raise.

We also noted the following points that need to be dealt with in the revised version.  
- The abstract, as it stands, doesn't really motivate the study.  You have only used about 165 words so you could include a couple of sentences of background, why the study is important to do and a clear concluding statement.
- We assume that the monkeys were used for in vivo analyses before the 'slices' of tissue were removed.  If this is the case, it needs to be clearly stated.  If it is not the case, or not the case for all monkeys, then a clear statement of the ethical issues around the killing of a monkey for this type of analysis is required e.g. they were going to be put down.
- You need to include details of what happened to the monkeys after the slice was removed.
- Please explicitly state that the work complied with EU regulations. (The EU 2010 directive).
- Please remove the 'Key points' section, include a COI statement and remove the extraneous text from journal names in the reference list
- Ensure that the reporting of statistical data adheres to EJN guidelines, notably report precise P values. Please also include statistical data within the text.
- Larger, higher resolution figures will be needed  for publication.

When revising the manuscript, please embolden or underline major changes to the text so they are easily identifiable and DO NOT leave 'track change' formatting marks in your paper.  Please ensure that you provide a text and a figure file for the Graphical Abstract (as detailed in the instructions below).  When carrying out your revisions please refer to the checklist below and visit the EJN author guidelines at www.ejneuroscience.org

When finalized, please upload your complete revised manuscript onto the website, as a Word file (.doc, or .docx).  Please also ensure that a complete set of tables and figures is included as separate files, even if these have not changed from the originals.  At this stage it is necessary to provide high resolution figures.  Please see important instructions below.

Please go into https://mc.manuscriptcentral.com/ejn - Author Centre - manuscripts with decisions where you will find a 'create a revision' link under 'actions'. We ask that you please indicate the way in which you have responded to the points raised by the Editors and Reviewers in a letter. Please upload this response letter as a separate Word (.doc or PDF) file using the file designation "Authors' Response to Reviewers" when uploading your manuscript files.  Please DO NOT submit your revised manuscript as a new one.  Also, please note that only the  Author who submitted the original version of the manuscript should submit a revised version.

If you are able to respond fully to the points raised, we would be pleased to receive a revision of your paper within 30 days.

Thank you for submitting your work to EJN.

Best wishes,

Paul Bolam & John Foxe
co-Editors in Chief, EJN


Reviews:

Reviewer: 1

Comments to the Author
The authors performed in vitro slice recording from the primary motor cortex of macaque monkeys.  They compared passive and active electrophysiological properties of presumably pyramidal neurons in superficial and deep cortical layers.  Both neurons showed bursting behaviors.  Deep layer neurons received excitatory inputs from all cortical layers, while superficial layer neurons received inputs from superficial layers.  This is a very unique report elucidating monkey cortical organization, which is different from rodents.  

Major
1) A diagram showing the arrangement of the recording neuron and the stimulating multiple-electrode will be useful.

2) Is there any morphological differences between superficial and deep neurons, or between bursting and regular spiking neurons?

3) Some histograms show two peaks, for example Figure 2D.  Do these two peaks correspond to bursting and regular spiking neurons, or fast and slow pyramidal tract  neurons?

4) Cortical stimulation may also stimulate thalamo-cortical fibers in addition to cortico-cortical fibers.  This should be discussed.

5) Do they record any IPSPs evoked by cortical stimulation?

Minor
1) Page 5 . Use i.v. instead of IV, such as propofol (i.v.) and i.v. infusion.

2)  Page 14, line 4.  Fig. 3E, not 3F.  


Reviewer: 2

Comments to the Author
Review of manuscript EJN-2018-04-25506
In Vitro Characterisation of Intrinsic Properties and Local Synaptic Inputs to Pyramidal Neurons in Macaque Primary Motor Cortex
Xu W, Baker SN

This study investigates the properties of pyramidal neurons in macaque motor cortex using intracellular slice recordings. This work is experimentally very challenging and has been performed on a large group of animals. The results are presented in a very concise way and organized around 4 main lines (i) the comparison of the membrane properties of the neurons (imput resistance, membrane time constant) recorded from the superficial and deep layers, (ii) the analysis of the action potential waveforms and the comparison between intracellular and extracellular recordings, (iii) the characterization of spiking activity in response to injected currents and (iv) the quantification of the neuronal responses to externally applied stimuli. Although the approach presented here is unique and provides highly valuable information for other experiments, the results sometimes appear over-interpreted to fit the author’s assumptions about the intrinsic organization of the macaque motor cortex.

For instance, based on the results presented in figure 2H and the suggestion that there is “considerable variability around the regression line”, the authors state that “extracellular parameters based on action potential shape are likely to be of only limited use in distinguishing between cells of different types in primate M1”.  This sounds like a harsh conclusion considering the rather convincing correlation shown in 2G and 2H suggesting a rather good relationship between the properties of intra and extracellular spikes. In addition, the data presented here include extracellular recordings of very variable amplitude (1.1mV to 17.2mV) which are likely to correspond to very different levels of signal-to-noise ratio. Would it be possible that spike waveform with low SNR deviate more from the regression line than “clean” spikes? This is somehow important for comparison purposes since many studies of extracellular spiking activity exclude waveforms with low SNR (usually below 2.5) from their analysis.

In the other way, based on the results presented in figure 4D-F, the authors favor the hypothesis of “a functionally near-unidirectional pathway from superficial to deep layers”. This sounds a rather strong statement based on the considerable variability of the stimulus depth evoking EPSPs in superficial neurons (figure 4E). In this respect, it is not entirely clear to me if all the stimulus-evoked responses (EPSPs or spikes) are monosynaptic in nature. The early EPSP in figure 4A is observed directly at the end of the stimulus and is followed by a second EPSP at longer latency (around 4 ms). The spike latency is around 2.5 ms, so quite late for a monosynaptic activation. Did the authors have a chance to analyze the latencies of the evoked responses in a systematic way? For instance, is there any latency difference between evokes EPSPs or spikes in the superficial and deep layers that could help to interpret their origin? In general, I find that the authors should clarify a bit more by which pathways the stimulus-evoked responses are carried out.

Minor:

Methods:
- The authors should provide more detailed information about the size and location of the block of tissue excised from the cortex. Is the excision performed at the laterality of the spur of the arcuate sulcus? On average, how many slices were obtained from each block. In terms of intracellular recordings, did the authors preferentially target the convexity or the depth of the central sulcus? These two parts of the motor cortex are likely to show substantial differences in their cellular and laminar organization.
- The two recording pipettes were moved simultaneously but it is not clear if they were always at a fixed distance from one another.
- In the analysis of the response to extracellular stimulation, the authors compare “the [peak] voltage at that time for each individual trial against the voltage value near the end of that trial”. As they point out, this approach creates a bias in the comparison that they attempt to correct by lowering the significance level to 1%. To be more consistent, the authors could compare the peak value within the first 50 ms post-stimulus to the peak value in the last 50 ms (i.e the 50 ms preceding the next stimulation).

Results
- 9 cells were stained with biocytin: could you specify how many of these cells were from the superficial and deep layers. The neuron presented in Figure 1A seems to be located in superficial layers.  Also, use a consistent terminology between the methods (Biocytin) and the results (Neurobiotin).
- Figure 4F could contain more information if it was a stacked histogram allowing to distinguish between spikes and EPSPs (i.e., it would indicate if most of the distribution comes from one or the other)

Typos
P5, materials and methods: “all experimental procedures were carried out under the authority…”
P7, Measurement of intrinsic properties: “by generating using semilogarithmic plots…”
P16, discussion: Do the authors refer to the below-described (and not above-described) unidirectional excitation from superficial to deep layers?

_____________________________________________________________________________________

Authors’ Response	20.06.2018

_____________________________________________________________________________________

Responses

Editor

- The abstract, as it stands, doesn't really motivate the study. You have only used about 165 words so you could include a couple of sentences of background, why the study is important to do and a clear concluding statement. A section has added at the start of the abstract describing the significance of the study.

- We assume that the monkeys were used for in vivo analyses before the 'slices' of tissue were removed. If this is the case, it needs to be clearly stated. If it is not the case, or not the case for all monkeys, then a clear statement of the ethical issues around the killing of a monkey for this type of analysis is required e.g. they were going to be put down.

Details added to Methods.

- You need to include details of what happened to the moneys after the slice was removed. Details added.

- Please explicitly state that the work complied with EU regulations. (The EU 2010 directive). Added

- Please remove the 'Key points' section, include a COI statement and remove the extraneous text from journal names in the reference list. Done.

- Ensure that the reporting of statistical data adheres to EJN guidelines, notably report precise P values. Please also include statistical data within the text. We have modified the text to include precise P values where previously we used ‘P<0.05’, and have added P values elsewhere where significance was merely stated.

- Larger, higher resolution figures will be needed for publication. We did submit .tif files at 600 dpi, at the final size expected to be used for publication, but also included lower resolution figures in the submitted Word file. We have now removed these lower resolution copies from the manuscript, leaving only the high resolution publication-quality figures.

Reviewer 1

Major

1) A diagram showing the arrangement of the recording neuron and the stimulating multipleelectrode will be useful. A schematic of the experimental setup has been inserted as an inset into Fig. 4A.

2) Is there any morphological differences between superficial and deep neurons, or between bursting and regular spiking neurons? Whilst all the labelled cells are without doubt pyramidal neurons, the relatively low numbers preclude us from making any meaningful conclusions about morphological parameters other than the obvious and trivial observation that deep pyramidal neurons have longer apical dendrites. All labelled neurons in our dataset happened to show bursting behaviour to current injection. We therefore do not have sufficient data to answer this interesting question.

3) Some histograms show two peaks, for example Figure 2D. Do these two peaks correspond to bursting and regular spiking neurons, or fast and slow pyramidal tract neurons? The overall histogram in figure 2D is not actually bimodal. The number of cells per interval is indicated by the highest point of the bin. Each bin is then split into two colours to indicate how many cells in that bin comes from superficial layer and how many from deep. This clearly needs to be explained better and we have amended the legend of Fig. 2. The histogram considering just deep cells is bimodal for Fig. 2D, as are the overall histograms for Figs 2C and 2E. In all cases there are neurons in both groups that show bursting to injected current. We have now added a comment to the text noting the bimodal distributions, and the lack of relationship to bursting.

It was not possible to identify pyramidal tract neurons in these in vitro experiments, and hence we cannot comment on whether cells did project to the pyramids, or their axonal conduction velocity.

4) Cortical stimulation may also stimulate thalamo-cortical fibers in addition to cortico-cortical fibers. This should be discussed. Consideration of the impact possible of thalamocortical projections has now been added to the Discussion.

5) Do they record any IPSPs evoked by cortical stimulation? We did not see any cells with a net inhibitory membrane response. That is not to say we have not activated inhibitory synapses as the summed response of EPSPs and IPSPs could still be a net excitation and slow IPSPs could influence the decay time constant of EPSPs without causing an overt hyperpolarisation. As we did not test the effect of pharmacological antagonists, it was not possible to separate excitation and inhibition. Therefore the inter-laminar excitations that we characterise are necessarily net excitations rather than pure excitations. We have added a comment at the start of the Results noting this finding.

Minor

1) Page 5 . Use i.v. instead of IV, such as propofol (i.v.) and i.v. infusion. Done

2) Page 14, line 4. Fig. 3E, not 3F. Done

Reviewer 2

Major

1) ... the authors state that “extracellular parameters based on action potential shape are likely to be of only limited use in distinguishing between cells of different types in primate M1”. This sounds like a harsh conclusion considering the rather convincing correlation shown in 2G and 2H suggesting a rather good relationship between the properties of intra and extracellular spikes.

The sentence has been modified to make it clear that although there is significant correlation, the degree of variability precludes the use of extra-cellular parameters alone in deriving intracellular waveforms.

2) In addition, the data presented here include extracellular recordings of very variable amplitude (1.1mV to 17.2mV) which are likely to correspond to very different levels of signal

Page 2 of 43European Journal of Neuroscience

For Peer Review

to-noise ratio. Would it be possible that spike waveform with low SNR deviate more from the regression line than “clean” spikes? This is somehow important for comparison purposes since many studies of extracellular spiking activity exclude waveforms with low SNR (usually below 2.5) from their analysis.

The reviewer makes a very valid point. To test this we separated the cells into two groups according to whether their extracellular AP size is above or below sample average. The correlation coefficient for cells below average size was actually higher than those above average for spike width and almost identical for peak/trough ratio. Therefore it seems that the variability in the relationships cannot be explained by the signal-to-noise ratio of the extracellular waveform. We have now added a paragraph to the Results describing this extra analysis.

3) In the other way, based on the results presented in figure 4D-F, the authors favor the hypothesis of “a functionally near-unidirectional pathway from superficial to deep layers”. This sounds a rather strong statement based on the considerable variability of the stimulus depth evoking EPSPs in superficial neurons (figure 4E).

The sentence has been altered in accordance with reviewer’s comments to make a less strong claim.

4) In this respect, it is not entirely clear to me if all the stimulus-evoked responses (EPSPs or spikes) are monosynaptic in nature. Did the authors have a chance to analyze the latencies of the evoked responses in a systematic way? In general, I find that the authors should clarify a bit more by which pathways the stimulus-evoked responses are carried out.

We have now added a section to the Discussion considering the probably pathways in more detail. For the reasons given there, we do not think that a detailed latency analysis is worthwhile.

Minor:

Methods: 1) The authors should provide more detailed information about the size and location of the block of tissue excised from the cortex. Is the excision performed at the laterality of the spur of the arcuate sulcus? These details have now been added to Methods.

2) On average, how many slices were obtained from each block. In terms of intracellular recordings, did the authors preferentially target the convexity or the depth of the central sulcus? These two parts of the motor cortex are likely to show substantial differences in their cellular and laminar organization. Details added to Methods. Recordings were always made from precentral gyrus.

3) In the analysis of the response to extracellular stimulation, the authors compare “the [peak] voltage at that time for each individual trial against the voltage value near the end of that trial”. As they point out, this approach creates a bias in the comparison that they attempt to correct by lowering the significance level to 1%. To be more consistent, the authors could compare the peak value within the first 50 ms post-stimulus to the peak value in the last 50 ms (i.e the 50 ms preceding the next stimulation). We initially tried an approach very similar to what the reviewer suggests, but found it to be too prone to the effects of noise. We found that on a trial-to-trial basis even if there exists an EPSP it is

Page 3 of 43 European Journal of Neuroscience

For Peer Review

not for certain that the highest voltage value in the first half of the trial would represent the EPSP peak – especially if the EPSP is small relative to background noise. The advantage of using the time of the averaged EPSP waveform peak is that one is more certain of where the EPSP peak is expected because the averaging removes noise. Furthermore if a stimulus contact is deemed not to produce a significant EPSP then we can be fairly certain that is the case, and not just because the data is noisy. It can be argued that in our study the contacts that don’t elicit a significant response are the interesting ones (e.g. the relative lack of response in superficial cells from deep stimuli), because all contacts can be made to elicit a response if the stimulus current is turned high enough.

4) The two recording pipettes were moved simultaneously but it is not clear if they were always at a fixed distance from one another. One electrode was placed in layer 2/3, the other in layer 5, so the distance along the slice was held fixed. They were advanced together through the slice until one found a cell. These details have now been added to the Methods.

5) 9 cells were stained with biocytin: could you specify how many of these cells were from the superficial and deep layers. The neuron presented in Figure 1A seems to be located in superficial layers. Also, use a consistent terminology between the methods (Biocytin) and the results (Neurobiotin). 4 deep, 5 superficial. Details added to Results section.

6) Figure 4F could contain more information if it was a stacked histogram allowing to distinguish between spikes and EPSPs (i.e., it would indicate if most of the distribution comes from one or the other) Done.

7) Typos P5, materials and methods: “all experimental procedures were carried out under the authority…” P7, Measurement of intrinsic properties: “by generating using semilogarithmic plots…” P16, discussion: Do the authors refer to the below-described (and not above-described) unidirectional excitation from superficial to deep layers? All corrected.

_____________________________________________________________________________________

2nd Editorial Decision	28.06.2018

_____________________________________________________________________________________

In Vitro Characterisation of Intrinsic Properties and Local Synaptic Inputs to Pyramidal Neurons in Macaque Primary Motor Cortex

Dear Dr. Xu,

Your revised manuscript was re-evaluated by external reviewers as well as by the Section Editor, Prof. John Foxe and ourselves.  We are pleased to inform you that it will be accepted for publication in EJN after you have dealt with a couple of minor points.  Please supply a list of abbreviations and a graphical abstract.  We also noted the typo 'paired ttest' on page 16.  Once done we will send it off to the publishers

When finalised, please upload your complete revised manuscript onto the website as a Word (.doc or .docx), or .rtf file.  Please also ensure that a complete set of tables and figures is included as separate files, even if these have not changed from the originals.  At this stage it is necessary to provide high resolution figures.  Please see important instructions below.

Please go into https://mc.manuscriptcentral.com/ejn - Author Centre - manuscripts with decisions where you will find a 'create a revision' link under 'actions'. We ask that you please indicate the way in which you have responded to the points raised by the Editors and Reviewers in a letter. Please upload this response letter as a separate Word (.doc) file using the file designation "Authors' Response to Reviewers" when uploading your manuscript files. Please DO NOT submit your revised manuscript as a new one.  Also, please note that only the  Author who submitted the original version of the manuscript should submit a revised version.

If you are able to respond fully to the points raised, we shall be pleased to receive a revision of your paper within 30 days.

Thank you for submitting your work to EJN.

Best wishes,

Paul Bolam & John Foxe
co-Editors in Chief, EJN

Reviews:

_____________________________________________________________________________________

Authors’ Response	15/06/2018

_____________________________________________________________________________________

Dear Editors:

Thank you for latest feedback on our paper ‘In Vitro Characterisation of Intrinsic Properties and Local

Synaptic Inputs to Pyramidal Neurons in Macaque Primary Motor Cortex’.

In accordance with your instructions we have now: - Added an abbreviations section – highlighted. - Corrected the typo ‘paired ttest’ on page 16. - Produced a Graphical abstract and accompanying text. - Produced high resolution .tiff files for figures and enlarged text within figures to improve

readability. - We have changed figure 2 into black-and-white in order to avoid colour figure printing costs.

Our funding body requires this study to be Open Access. Our institution’s library will meet the cost

of publishing Open Access in due course. Please let us know this fee is required.

Yours sincerely

Wei Xu

Stuart Baker
